# Supplementary material for: Quantifying DNA replication speeds in single cells by scEdU-seq
Source: Nat Methods. 2024 Jun 17;21(7):1175–84. doi: 10.1038/s41592-024-02308-4 (PMC11239516; doi:10.1038/s41592-024-02308-4)
Supplement: Supplementary file 1 — Reporting Summary [file 41592_2024_2308_MOESM1_ESM.pdf]

Corresponding author(s): Alexander van Oudenaarden & Jeroen van den Berg

Last updated by author(s): Apr 22, 2024

Reporting Summary

Nature Portfolio wishes to improve the reproducibility of the work that we publish. This form provides structure for consistency and transparency in reporting. For further information on Nature Portfolio policies, see our Editorial Policies and the Editorial Policy Checklist.

Statistics

For all statistical analyses, confirm that the following items are present in the figure legend, table legend, main text, or Methods section.

n/a	Confirmed	
☐	☒	The exact sample size (*n*) for each experimental group/condition, given as a discrete number and unit of measurement
☐	☒	A statement on whether measurements were taken from distinct samples or whether the same sample was measured repeatedly
☐	☒	The statistical test(s) used AND whether they are one- or two-sided<br>*Only common tests should be described solely by name; describe more complex techniques in the Methods section.*
☐	☒	A description of all covariates tested
☐	☒	A description of any assumptions or corrections, such as tests of normality and adjustment for multiple comparisons
☐	☒	A full description of the statistical parameters including central tendency (e.g. means) or other basic estimates (e.g. regression coefficient) AND variation (e.g. standard deviation) or associated estimates of uncertainty (e.g. confidence intervals)
☐	☒	For null hypothesis testing, the test statistic (e.g. *F*, *t*, *r*) with confidence intervals, effect sizes, degrees of freedom and *P* value noted<br>*Give P values as exact values whenever suitable.*
☐	☒	For Bayesian analysis, information on the choice of priors and Markov chain Monte Carlo settings
☒	☐	For hierarchical and complex designs, identification of the appropriate level for tests and full reporting of outcomes
☒	☐	Estimates of effect sizes (e.g. Cohen's *d*, Pearson's *r*), indicating how they were calculated

Our web collection on statistics for biologists contains articles on many of the points above.

Software and code

Policy information about availability of computer code

Data collection

Sequencing data were collected on the Illumina NextSeq 500 or 2000 (NextSeq Control Software version 2.2.0.4), using standard software for basecalling (RTA version 2.4.11). Sample demultiplexing was performed using bcl2fastq (v2.20.0.422).

FACS data were collected on a BD Influx (BD FACS Sortware version 1.2.0.142) for sorting experiments

FACS data were collected on a Beckman-Coulter Cytoflex (CytExpert software version 2.5, March 2022) for flow cytometry experiments

Western Blot development was performed on a Bio-Rad Chemi-Doc MP with manufacturers software

Data analysis

Data were analyzed using a combination of publicly available and custom software.
Publicly available software included: SingleCellMultiOmics (v0.1.2.5), cutadapt (version 3.2), bwa (version 2.7.6a), python (versions 3.7.3 & 3.8.2), round_0.20-0, umap_0.2.8.0, mhsmm_0.4.16, mvtnorm_1.1-3, flexmix_2.3-17, lattice_0.20-45, Rcpp_1.0.7, forcats_0.5.1, stringr_1.4.0, dplyr_1.0.9, purrr_0.3.4, readr_2.1.2, tidyr_1.2.0, tibble_3.1.7, ggplot2_3.3.6, tidyverse_1.3.1, data.table_1.14.2, R version 4.2.0 (2022-04-22)
All custom scripts to process raw data and generate figures are available at https://github.com/vincentvbatenburg/scEdU-seq

For manuscripts utilizing custom algorithms or software that are central to the research but not yet described in published literature, software must be made available to editors and reviewers. We strongly encourage code deposition in a community repository (e.g. GitHub). See the Nature Portfolio guidelines for submitting code & software for further information.

Data

Policy information about availability of data

All manuscripts must include a data availability statement. This statement should provide the following information, where applicable:

- Accession codes, unique identifiers, or web links for publicly available datasets
- A description of any restrictions on data availability
- For clinical datasets or third party data, please ensure that the statement adheres to our policy

Raw sequencing data, metadata and count tables have been made available in the Gene Expression Omnibus under the accession number GSE211037.

Data for comparisons to scEUseq and scRepli-Seq were downloaded from Gene Expression Omnibus accessions GSE128365 and GSE108556. Raw sequencing data of DNA replication origins was downloaded from SRA (PRJNA397123). Data for Replication timing was downloaded from the 4D nucleome project (4DNBSKYMY5XL)

Human research participants

Policy information about studies involving human research participants and Sex and Gender in Research.

Reporting on sex and gender	n/a
Population characteristics	n/a
Recruitment	n/a
Ethics oversight	n/a

Note that full information on the approval of the study protocol must also be provided in the manuscript.

Field-specific reporting

Please select the one below that is the best fit for your research. If you are not sure, read the appropriate sections before making your selection.

☒ Life sciences ☐ Behavioural & social sciences ☐ Ecological, evolutionary & environmental sciences

For a reference copy of the document with all sections, see nature.com/documents/nr-reporting-summary-flat.pdf

Life sciences study design

All studies must disclose on these points even when the disclosure is negative.

Sample size	No statistical methods were used to predetermine sample size. The number of cells analyzed was chosen to enable sufficient technical validation and benchmarking of scEdUseq. A minimum of 100 cells per replicate was used to enable proper ordering of cells along S-phase.
Data exclusions	All raw data are uploaded in public repositories.<br>In downstream analyses, cells were excluded if they did not meet two quality control thresholds: a minimum number of average reads per bin per cell and a coefficient of variation clearly deviating from Poisson noise. These thresholds removed cells where the library construction and/or EdU incorporation failed. The values of these cutoffs were slightly adjusted for each plate and sample type to compensate for differences in sequencing depth and EdU incorporation.
Replication	For the hTERT-RPE-1 cell-cycle experiments, the DMSO and PARP treated cells were measured in three independent experiments, and the DRB and XRCC1 experiments were measured in one experiment. All attempts at replication were successful.
Randomization	No randomization was performed. Randomization was not necessary as all samples were processed in an automarted fashion by robotic liquid handlers.
Blinding	No blinding was performed since we performed unsupervised analysis techniques (e.g., clustering and dimensionality reduction)

Reporting for specific materials, systems and methods

We require information from authors about some types of materials, experimental systems and methods used in many studies. Here, indicate whether each material, system or method listed is relevant to your study. If you are not sure if a list item applies to your research, read the appropriate section before selecting a response.

Materials & experimental systems

n/a	Involved in the study
☐	☒ Antibodies
☐	☒ Eukaryotic cell lines
☒	☐ Palaeontology and archaeology
☒	☐ Animals and other organisms
☒	☐ Clinical data
☒	☐ Dual use research of concern

Methods

n/a	Involved in the study
☒	☐ ChIP-seq
☐	☒ Flow cytometry
☒	☐ MRI-based neuroimaging

Antibodies

Antibodies used	XRCC1 (abcam, ab1838) (1:1000)<br>CDK4 (Santa Cruz Biotechnologies, sc-260) (1:1000)<br>pan ADP-ribose (MABE1016, Sigma-Aldrich) (1:1500)<br>gH2AX (Millipore, JBW301) (1;!000)<br>anti-H3K9Me3 (Abcam, ab8898, 1:100),<br>anti-H3K27Me3 (Cell Signaling Technologies, C36B11, 1:200) and<br>anti-H3K36Me3 (Thermo Fisher, MA5-24687, 1:2000).
Validation	XRCC1 (https://www.abcam.com/xrcc1-antibody-33-2-5-ab1838.html), Hoch et al., Nature 2017. CDK4 (https://www.scbt.com/p/cdk4-antibody-c-22), van den berg et al., NAR, 2016. pan ADP-ribose (https://www.merckmillipore.com/NL/en/product/Anti-pan-ADP-ribose-binding-reagent,MM_NF-MABE1016) Hoch et al., Nature 2017. gH2AX (https://www.merckmillipore.com/NL/en/product/Anti-phospho-Histone-H2A.X-Ser139-Antibody-clone JBW301,MM_NF-05-636) van den Berg et al., NAR, 2016 anti-H3K9Me3 (Abcam, ab8898, 1:100), Zeller et al., Nature Genetics 2022, anti-H3K27Me3 (Cell Signaling Technologies, C36B11, 1:200) Zeller et al., Nature Genetics 2022, and anti-H3K36Me3 (Thermo Fisher, MA5-24687, 1:2000) Zeller et al., Nature Genetics 2022.

Eukaryotic cell lines

Policy information about cell lines and Sex and Gender in Research

Cell line source(s)	hTERT RPE-1 FUCCI and hTERT RPE-1 iCut cells were both obtained from the Medema lab at the Netherlands Cancer Institute.
Authentication	None of the cell lines were authenticated.
Mycoplasma contamination	All cell lines routinely tested negative for Mycoplasma contamination
Commonly misidentified lines<br>(See ICLAC register)	No commonly misidentified cell lines were used in the study.

Flow Cytometry

Plots

Confirm that:

☒ The axis labels state the marker and fluorochrome used (e.g. CD4-FITC).

☒ The axis scales are clearly visible. Include numbers along axes only for bottom left plot of group (a 'group' is an analysis of identical markers).

☒ All plots are contour plots with outliers or pseudocolor plots.

☒ A numerical value for number of cells or percentage (with statistics) is provided.

Methodology

Sample preparation	hTERT RPE-1 FUCCI cells: Cells were dissociated to a single-cell suspension using TrypLE, washed and resuspended in PBS containing DAPI as a viability stain and BSA to reduce aggregation. Cells were passed through a 20-micron mesh before sorting.
Instrument	BD Influx and Beckman-Coulter Cytoflex
Software	BD FACS Sortware 1.2.0.142 and CytExpert software
Cell population abundance	Cell population purity and abundance was not explicitly determined after sorting. FACS was primarily used to i) distribute single cells into individual wells of a 384-well plate for subsequent processing, ii) measure the fluorescence of cell-cycle progression markers of these cells,.
Gating strategy	hTERT RPE-1 Doublets, debris, and dead cells were excluded by gating forward and side scatter in combination with

Gating strategy

the DAPI channel. Example gating strategy for is provided in Extended Data Figures 1a and 1c.

☒ Tick this box to confirm that a figure exemplifying the gating strategy is provided in the Supplementary Information.
